# Dr. Horatio Gates Hall

**Published:** 1901-08

**Authors:** 


### DR. HORATIO GATES HALL.

AT his residence, in Piqua, Ohio, Dr. Horatio Gates Hall died, May 23, 1901, of Bright's disease, aged seventy-one years.

Dr. Hall was one of the best known dentists in Western Ohio. He had been actively engaged in the practice of dentistry for forty-six years. He was an honorable, genial, and Christian gentleman, and one whose acquaintance and friendship was well worth seeking. He leaves a wife, two daughters, and two sons, and is the first of four brothers, all dentists, to be claimed by death. He will be greatly missed.

